# The effects of probiotics intervention on oral health outcomes: a comprehensive umbrella review of meta-analyses

**DOI:** 10.3389/froh.2026.1768508

**Published:** 2026-03-26

**Authors:** Zhen Tang, Yunzhen Deng, Gang Guo, LiLi Zhou

**Affiliations:** 1 Teaching and Research Section of Oral and Maxillofacial Surgery, Fuzhou Medical College, Fuzhou, China; 2 State Key Laboratory of Military Stomatology & National Clinical Research Center for Oral Diseases & Shaanxi International Joint Research Center for Oral Diseases, Department of General Dentistry and Emergency, School of Stomatology, Fourth Military Medical University, Xi'an, China; 3 Center for Gut Microbiome Research, Med-X Institute Centre, First Affiliated Hospital of Xi’an Jiao Tong University, Xi'an, China

**Keywords:** dental caries, halitosis, lactobacillus, oral health, probiotics, streptococcus, umbrella review

## Abstract

**Background:**

Probiotics are proposed adjuncts for oral health, but findings from meta-analyses are inconsistent. We conducted an umbrella review of meta-analyses to synthesize evidence on probiotics’ effects on cariogenic bacteria such as *Streptococus (S.) mutans* and *Lactobacillus*, halitosis (volatile sulfur compounds and organoleptic scores), and caries progression.

**Methods:**

PubMed, Web of Science and Scopus databases were searched up to October 2025 for meta-analyses of clinical trials investigating the effect of probiotics on oral health outcomes. Two reviewers screened studies, extracted data on strains, doses, durations, and pooled effect estimates, and assessed review quality using AMSTAR-2. Directions and key pooled estimates reported in included meta-analyses were summarized.

**Results:**

Eleven meta-analyses met inclusion criteria, encompassing participants from infancy to adulthood (reported mean/median ages 0.2–42.5 years), probiotic doses reported between ∼5 × 10^5^ and 5 × 10^10^ CFU, and intervention durations of 7 days to 24 months. Common strains included *Lactobacillus*, *Bifidobacterium*, and *S. salivarius*. Probiotics were associated with reductions in *S. mutans* counts in most reported comparisons and improved halitosis measures in the majority of comparisons. Pooled estimates for caries-related outcomes indicated modest reductions (example pooled SMDs reported∼−0.24; 95% CI −0.39 to −0.10 and for *Lactobacillus rhamnosus* −0.41; 95% CI −0.60 to −0.21). Most meta-analyses were rated moderate by AMSTAR-2; substantial heterogeneity, variable strains/delivery, and reliance on surrogate endpoints were common.

**Conclusions:**

Evidence suggests modest benefits of certain probiotic strains on cariogenic bacteria and halitosis, but heterogeneity, surrogate outcomes, and moderate review quality limit certainty. High-quality, strain-specific RCTs with standardized clinical endpoints are needed.

## Introduction

Oral health plays a crucial role in overall well-being and quality of life. Beyond chewing, the oral cavity acts as a complex ecosystem where microbial, host, and environmental factors continuously interact (1). Disturbances in this balance can contribute oral diseases such as dental caries, periodontitis, and halitosis, which could cause important personal and public health burdens. Halitosis, or chronic bad breath, one of the most common oral complaints, is mainly linked to the oral microbiome. This condition affects roughly one-third of people worldwide, with reported rates ranging from as low as 2.4% to as high as 55%, depending on how it is defined and measured (2). Halitosis can be classified into intraoral and extraoral types, with the intraoral form responsible for approximately 80%–90% of all cases (3). It occurs when certain microbes break down protein-rich substances and release volatile sulfur compounds (VSCs) like methyl mercaptan (CH₃SH), hydrogen sulfide (H₂S), and dimethyl sulfide [(CH₃)₂S] (4). The back of the tongue, in particular, tends to accumulate debris and harbor bacteria, creating ideal conditions for the production of VSCs. On the other hand, *Streptococcus* (*S.*) *mutans* and *Lactobacillus* are key bacterial groups influencing oral health. Measuring their levels is commonly used to assess caries risk and evaluate the balance of the oral microbiome (5). High levels of *S. mutans* are associated with the initiation of dental caries, whereas *Lactobacillus* is more closely linked to dentinal involvement.

Probiotics are beneficial, non-pathogenic microorganisms that help maintain microbial balance, thereby contributing positively to overall health (6). Probiotic supplementation appears to influence oral health by modulating organic malodor, microbial counts, and inflammatory responses, with implications for halitosis, and shifts in *Streptococcus* and *Lactobacillus* populations (7, 8). Shirbhate et al. demonstrated that probiotics can effectively compete with pathogens, produce antimicrobial substances or hydrogen peroxide, and modulate host immunity to reduce inflammation. Studies demonstrated that microorganisms such as *Fusobacterium nucleatum*, *Porphyromonas gingivalis*, *Prevotella intermedia*, *Prevotella nigrescens*, and *Treponema denticola* are associated with periodontal diseases and may also contribute to the production of VSCs (7, 9). For this reason, maintaining a balanced oral microbiota level plays a crucial role in managing halitosis. Furthermore, Patil et al. found that short-term topical or local use of probiotics can significantly lower *S. mutans* levels in dental plaque. In a trial involving caries-active children aged 7–12, applying a probiotic formulation for six consecutive days reduced plaque *S. mutans* counts from approximately 608,000 to 6,600 CFU, a reduction of over 90% compared to baseline (10).

Nevertheless, the certainty of the current evidence remains low to moderate. Most available trials are short in duration and differ widely in the probiotic strains used, dosing regimens, and delivery vehicles. As a result, findings across individual studies and meta-analyses are often inconsistent and difficult to interpret in isolation. Given the growing number of meta-analyses on probiotics and oral health, there is a clear need for an umbrella review to bring this evidence together and provide a more coherent overview. Accordingly, this study synthesizes and compares existing meta-analyses examining the effects of probiotics on halitosis, *Streptococcus* mutans, and *Lactobacillus* counts.

## Methods

This umbrella systematic review adhered to the guidelines outlined in the Preferred Reporting Items for Systematic Reviews and Meta-analysis (PRISMA) (11), ensuring a structured and rigorous approach throughout the study process.

### Search strategy

A thorough search was conducted across international scientific databases such as PubMed, Scopus, EMBASE, and Web of Science, to identify pertinent articles. Our search encompassed articles from the inception of each database up to October 2025. To refine our search, we employed a strategy incorporating MeSH terms and keywords: (((((“oral health"[MeSH Terms]) OR (“oral flora"[MeSH Terms])) OR (“dental caries"[MeSH Terms])) OR (caries, dental[MeSH Terms])) OR (oral hygiene[MeSH Terms])) OR (((((“oral health"[Title/Abstract]) OR (“Oral flora"[Title/Abstract])) OR (“dental caries"[Title/Abstract])) OR (caries[Title/Abstract])) OR (“Oral hygiene"[Title/Abstract])) OR (dental plaque[Title/Abstract])) AND ((((“Probiotics"[MeSH]) OR (Bifidobacteria[Title/Abstract])) OR (lactobacilli[Title/Abstract])) OR (Probiotics[Title/Abstract])) AND meta-analysis[Title/Abstract].

Additionally, we restricted our search to articles published exclusively in English. The utilization of the wildcard term “*” bolstered the sensitivity of our search strategy.

### Inclusion and exclusion criteria

The present umbrella review incorporated meta-analysis studies examining the effect of probiotics on dental caries and halitosis. Studies were excluded if they were *in vitro, in vivo*, and *ex vivo* studies, case reports, *quasi*-experimental studies, randomized controlled trials (RCTs) and low-quality meta-analyses. The PICO criteria for this umbrella systematic review were as follows: population (children and adults with normal or impaired oral health), the intervention (oral probiotic supplementation), the comparators (placebo, no intervention, or standard oral care), and the outcomes (*Streptococcus* and *Lactobacillus* counts, halitosis measures, and caries incidence or progression).

### Methodological quality assessment

Two reviewers, (ZT, YD), independently evaluated the methodological rigor of the included articles using the Assessing the Methodological Quality of Systematic Reviews 2 (AMSTAR2) questionnaire. In instances of disagreement, the senior author (LZ) was consulted to reconcile and achieve a consensus. The AMSTAR2 questionnaire comprises 16 items, each of which is assessed with responses such as “Yes”, “Partial Yes”, “No”, or “Not a Meta-analysis”. The assessment outcomes were categorized into “Critically low quality”, “Low quality”, “Moderate quality”, and “High quality”, based on the AMSTAR2 checklist criteria.

### Study selection and data extraction

Two independent reviewers (ZT and YD) conducted the initial screening of articles for eligibility. This screening process involved reviewing the title and abstract of each article, followed by a thorough assessment of the full text of relevant articles to determine their suitability for inclusion in the umbrella meta-analysis. Any discrepancies between the reviewers were resolved through consensus with a third reviewer (LZ). Data pertaining to various aspects including the year of publication, sample size, study type, study location, journal of publication, type of study complication, health condition, type of treatment, probiotic species, doses and duration range of supplementation, effect sizes (ESs), and corresponding confidence intervals (CIs) for dental caries and halitosis were extracted from the selected meta-analyses and recorded in an Excel spreadsheet.

## Results

### Study selection

**Figure 1:**
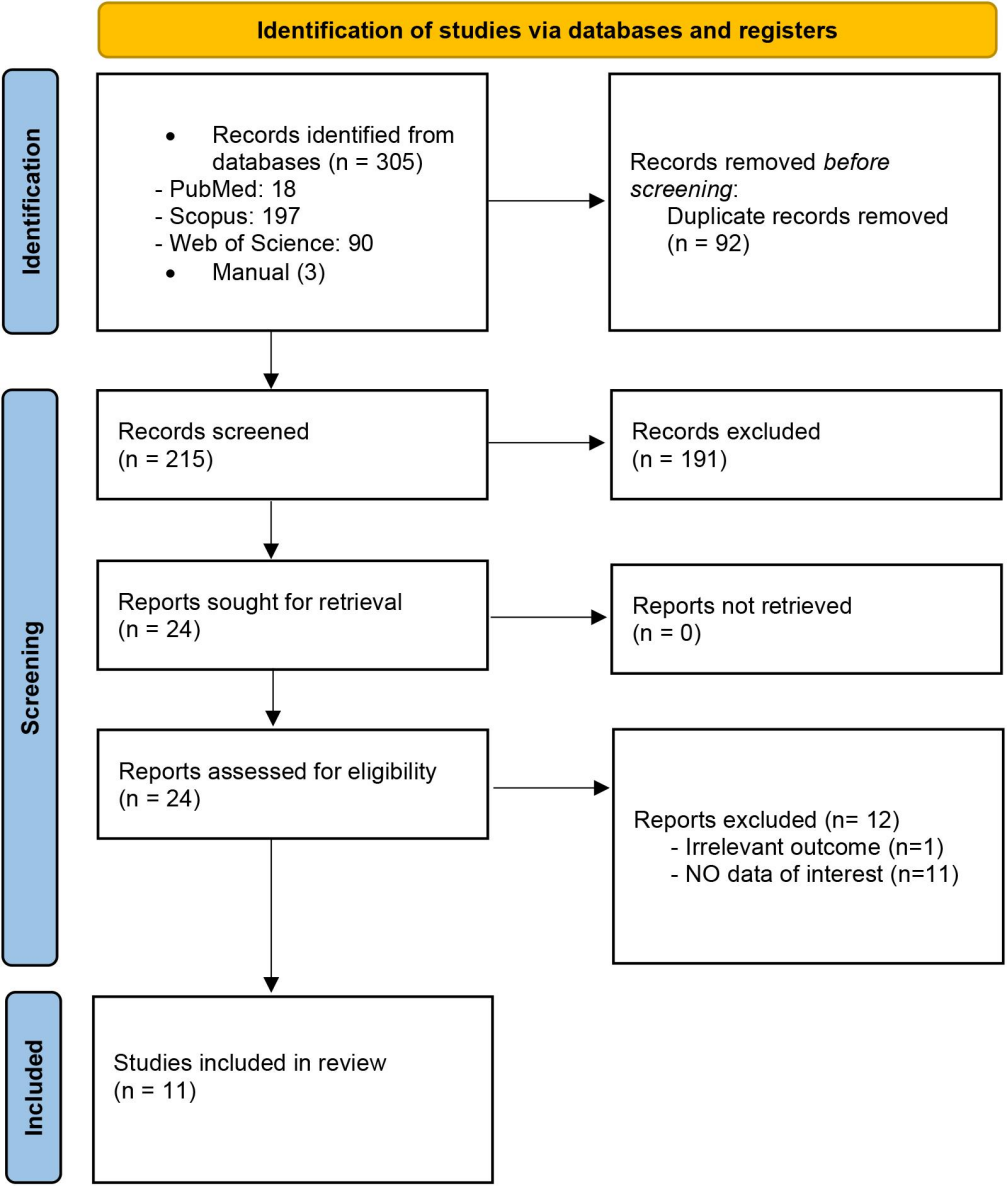
PRISMA flowchart of the study showing the study selection process.

The present study utilized meta-analyses of controlled trials. Initially, a total of 308 articles were identified through a comprehensive search of the database. After removing duplicates, 215 unique studies were screened based on their title and abstract. Following a thorough examination of the titles and abstracts, reports excluded refer to full-text articles that were assessed for eligibility but excluded after full-text review because they did not meet the inclusion criteria (e.g., not a meta-analysis, irrelevant outcomes, or insufficient data), 11 studies were deemed relevant and subsequently included in the study. The flow diagram illustrating the literature search process is summarized in Figure 1.

### Study characteristics

The characteristics of included studies are presented in Table 1. The age of studies’ participants was between 0.2 and 42.5 years. In total, dose varied between 5 × 10^5^ and 5 × 10^10^. Moreover, duration varied between 7 days to 24 months.

**Table 1:** Characteristics of the included meta-analyses in the umbrella review.

Author name/Year	Location	No.of study	No. of participant	Subgroups	Age category (age mean[years])	Type of probiotic	Dose -Mean(CFU)/Duartion-Mean	Main finding
He (12)/2023	China	33	60 to 7,422	Dental caries (incidence/progression)	Preschool children up to 72 months old(NR)	Probiotic milk plus low fluoride toothpaste	NR/NR	Probiotic milk plus low fluoride toothpaste were more effective in preventing caries incidence compared to other treatment.
Meng (13)/2023	China	10	1,951	Dental caries (incidence/progression)	Children (3)	* Lactobacillus, Bifidobacterium, Streptococcus*	10.2 × 10^8^/13.3M	probiotics effectively prevent dental caries Lactobacillus rhamnosus was more effective/reduction the high concentration of Streptococcus mutans in saliva but not the number of lactic acid bacteria in saliva and dental plaques
		8	1,449	Dental caries (incidence/progression)	Children (3)	* Lactobacillus, Bifidobacterium, Streptococcus*	3.2 × 10^7^/9.6M	
		6	1,748	Strepto count	Children (3)	* Lactobacillus, Bifidobacterium*	5 × 10^7^/8M	
		2	589	Lacto count	Children (3.5)	* Lactobacillus, Bifidobacterium*	5 × 10^7^/8M	
Hao (14)/2021	China	8	345	Strepto count	Children and youth (19)	* Bifidobacterium*	1.8 × 10^10^/13D	Bifidobacterium was ineffective in reducing Streptococcus mutans and Lactobacillus counts in the saliva or dental plaque/Not able to inhibit the initiation of deciduous dental caries
		11	345	Lacto count	Children and youth (19)	* Bifidobacterium*	1.8 × 10^10^/13D	
Chen (15)/2023	China	3	227	Strepto count	Children, youth and adults (15)	* Lactobacillus, Bifidobacterium, Streptococus*	1.2 × 10^10^/26D	potential benefits probiotics in reducing salivary strepto counts
		3	196	Lacto count	Children, youth and adults (15)	* Lactobacillus, Bifidobacterium*	1.5 × 10^8^/32D	
Shi (16)/2022	China	16	1,962	Strepto count	Children(7.5)	* Lactobacillus, Bifidobacterium Streptococcus*	6.6 × 10^10^/5M	probiotics presented a favorable effect in controlling dental caries, salivary S. mutans and Lactobacillus counts in children.
		8	831	Lacto count	Children(7.8)	* Lactobacillus*	10^10^/4M	
Huang (7)/2022	China	5	200	Halitosis (OLP)	Adult(32)	* Lactobacillus, Weissella cibaria, Streptococcus*	1.3 × 10^9^/ <4M	probiotics may ease halitosis by reducing the VSC concentration in the short term/there is no significant effect on the major cause of halitosis such as plaque and tongue coating
		6	254	VSC	Adult(33)	* Lacto bacillus, Weissella cibaria, Streptococcus*	6 × 10^8^/ <4M	
		2	97	OLP	Adult(35.5)	* Lactobacillus, Weissella cibaria*	2 × 10^9^/ > 4M	
		2	126	VSC	Adult(37)	* Weissella cibaria*	10^8^/ > 4M	
Gruner (17)/2016	Germany	19	1,030	Strepto count	Children(NR)	*NR*	1.26 × 10^12^/NR	Insufficient evidence for recommending probiotics for managing dental caries
		14	724	Lacrobacilli count	Children(NR)	*NR*	1.26 × 10^12^/NR	
Valverde (18)/2022	Spain	4	289	Halitosis	6–44 years old (22.7)	* Weissella cibaria, Streptococcus*	NR/2.1M	Some probiotics have a beneficial effect on halitosis
Yoo (19)/2017	Korea	3	104	Halitosis (OLP)	Adult (42.5)	* Lactobacillus*	3 × 10^9^/42D	Effectiveness of probiotics for the management of halitosis/Lactobacilli were more effective.
		3	106	VSC	Adult (29.8)	* Lactobacillus*	2.5 × 10^8^/14D	
		6	201	Total	Adult(36.2)	* Lactobacillus*	1.6 × 10^9^/28D	
Tovalino (5)/2024	Peru	11	1,321	Strepto count	Children and adolescents(15)	* Lactobacillus/Bifidobacterium*	1 × 10^9^/4 M	Probiotics likely reduce S. mutans levels, which are strongly linked to caries risk, but do not significantly affect Lactobacillus levels. Effects appear strain- and formulation-dependent.
		6	1,134	Lacto count	Children and adolescents(15)	* Lactobacillus/Bifidobacterium*	1 × 10^9^/5 M	
Offenbächer (4)/2024	Spain	3	280	Halitosis(OLP)	Adults (28.2)	* Lactobacillus/Streptococcus/Weissella species*	5 × 10^8^/2 M	Probiotic supplementation for 4 weeks led to a significant decrease in (VSC) concentrations and improved OLP scores, demonstrating a measurable reduction in halitosis severity. Lactobacillus-based formulations were more active.
		4	300	VSC	Adults (27.6)	* Lactobacillus/Streptococcus/Weissella species*	5 × 10^8^/1.8M	

Reported characteristics reflect information available in the original meta-analyses; missing data are indicated as NR (not reported), D, day; M, month; OLP, organoleptic; VSC, volatile sulphur compounds.

Six studies were conducted in China (7, 12–16), one in Germany (17), two in Spain (4, 8), one in Peru (5) and one in Korea (19). Totally, 4 studies with 9 effect sizes were about *Streptococcus* count (13–16), while 4 articles with 8 effect size were about *Lactobacillus* count (13–16), 3 studies with 8 effect sizes were about halitosis (7, 8, 19).

### Risk of bias assessment

The majority of the included meta-analyses in the umbrella review were assessed as being of moderate quality. The outcomes of the quality assessment of meta-analyses based on the AMSTAR2 questionnaire are presented in Table 2.

**Table 2:** Quality assessment of the included studies based on AMSTAR2 questionnaire.

First author	Q1	Q2	Q3	Q4	Q5	Q6	Q7	Q8	Q9	Q10	Q11	Q12	Q13	Q14	Q15	Q16	Overall
He et al.	Yes	P.Y	Yes	Yes	Yes	Yes	Yes	No	P.Y	Yes	Yes	No	No	No	Yes	Yes	Moderate
Meng et al.	Yes	No	Yes	Yes	Yes	Yes	No	Yes	Yes	Yes	Yes	Yes	No	No	No	Yes	Moderate
Hao et al.	No	No	Yes	P.Y	Yes	Yes	No	No	P.Y	Yes	Yes	No	No	Yes	No	Yes	Moderate
Chen et al.	Yes	Yes	Yes	P.Y	Yes	Yes	P.Y	Yes	Yes	No	Yes	No	No	Yes	No	Yes	Moderate
Shi et al.	Yes	No	Yes	P.Y	No	No	P.Y	P.Y	Yes	No	Yes	Yes	No	Yes	No	Yes	Low
Huang et al.	Yes	P.Y	Yes	P.Y	Yes	Yes	Yes	P.Y	Yes	Yes	Yes	Yes	No	No	Yes	Yes	High
Gruner et al.	No	Yes	Yes	P.Y	Yes	Yes	P.Y	Yes	No	No	Yes	No	Yes	Yes	No	No	Low
Valverde et al.	Yes	Yes	Yes	P.Y	Yes	Yes	Yes	P.Y	P.Y	No	Yes	Yes	No	Yes	No	Yes	Moderate
Yoo et al.	Yes	P.Y	Yes	P.Y	Yes	Yes	Yes	P.Y	Yes	No	Yes	Yes	Yes	Yes	No	Yes	Moderate
Tovalino et al.	Yes	Yes	Yes	P.Y	Yes	Yes	P.Y	Yes	Yes	No	Yes	No	No	Yes	No	Yes	Moderate
Offenbächer et al	Yes	No	Yes	P.Y	No	No	P.Y	P.Y	Yes	No	Yes	Yes	No	Yes	No	Yes	Low

PY, partial yes.

Q1- Did the research questions and inclusion criteria for the review include the components of PICO? Q2- Did the report of the review contain an explicit statement that the review methods were established prior to the conduct of the review, and did the report justify any significant deviations from the protocol? Q3- Did the review authors explain their selection of the study designs for inclusion in the review? Q4- Did the review authors use a comprehensive literature search strategy? Q5- Did the review authors perform study selection in duplicate? Q6- Did the review authors perform data extraction in duplicate? Q7- Did the review authors provide a list of excluded studies and justify the exclusions? Q8- Did the review authors describe the included studies in adequate detail? Q9- Did the review authors use a satisfactory technique for assessing the risk of bias (RoB) in individual studies that were included in the review? 10- Did the review authors report on the sources of funding for the studies included in the review? Q11- If meta-analysis was performed, did the review authors use appropriate methods for the statistical combination of results? Q12- If a meta-analysis was performed, did the review authors assess the potential impact of RoB in individual studies on the results of the meta-analysis or other evidence synthesis? Q13- Did the review authors account for RoB in individual studies when interpreting/discussing the review results? Q14- Did the review authors provide a satisfactory explanation for and discussion of any heterogeneity observed in the review results? Q15- If they performed quantitative synthesis, did the review authors conduct an adequate investigation of publication bias (small-study bias) and discuss its likely impact on the review results? Q16- Did the review authors report any potential sources of conflict of interest, including any funding they received for conducting the review?

### The effects of probiotics supplementation on overall *Streptococcus* count

Five eligible studies with 10 effect sizes [6 risk ratio (RR) and 4 Standardized mean difference (SMD)], including 6,767 participants, examined the impact of probiotics supplementation on overall *Streptococcus* count. Eight of them demonstrated a significant favorable effect on *Streptococcus* count. The age varied between 3 and 20 years. The primary strains that were supplemented included *Lactobacillus*, *Bifidobacterium*, and *Streptococcus*. The effect sizes of studies with CI are presented in Table 3.

**Table 3:** The reported effect sizes and confidence intervals for each outcome based on subgroup analyses.

Author name	Subgroup	Type of effect size (number of included observations)	Effect size	(LCI, UCI)
Meng	*Lactobacillus, Bifidobacterium, Streptococcus*	RR(10)	0.7	(0.54, 0.91)
Meng	*Lactobacillus paracasei*	RR(2)	0.82	(0.45, 1.5)
Meng	*Lactobacillus rhamnosus*	RR(4)	0.62	(0.45, 0.87)
Meng	*Bifidobacterium*	RR(2)	0.86	(−3.05, −1.22)
Dental caries (incidence) (dmft/DMFT or dmfs/DMFS scores, incidence of new carious lesions)				
Meng	*Lactobacillus, Bifidobacterium, Streptococcus*	SMD(8)	−0.24	(−0.39, −0.1)
Meng	*Lactobacillus paracasei*	SMD(3)	−0.16	(−0.33, 0.01)
Meng	*Lactobacillus rhamnosus*	SMD(3)	−0.41	(−0.6, −0.21)
Streptococcus count				
Hao	*Bifidobacterium*	SMD(4)	−0.32	(−0.67, 0.04)
Hao	*Bifidobacterium*	RR(4)	0.53	(0.17, 1.66)
Chen	*Lactobacillus, Bifidobacterium, Streptococcus, S mutans <10^5^*	RR(3)	2.05	(1.54, 2.72)
Chen	*Lactobacillus, Bifidobacterium, Streptococcus S mutans >10^5^*	RR(3)	0.48	(0.28, 0.83)
Meng	*Lactobacillus, Bifidobacterium*	SMD(2)	−0.16	(−0.33, 0.01)
Meng	*Lactobacillus*	RR(4)	0.62	(0.51, 0.74)
Shi	*Lactobacillus, Bifidobacterium*	SMD(10)	−1.17	(−1.85, −0.5)
Shi	*Lactobacillus, Bifidobacterium, Streptococcus S mutans <10^5^*	RR(6)	0.63	(0.5, 0.8)
Shi	*Lactobacillus, Bifidobacterium, Streptococcus S mutans >10^5^*	RR(6)	1.6	(1.1, 2.34)
Tovalino	–	SMD(11)	−0.4	(−0.57, −0.24)
Lacto count				
Hao	*Bifidobacterium*	SMD(8)	−0.07	(−0.39, 0.26)
Hao	*Bifidobacterium*	RR(3)	0.87	(0.59, 1.29)
Chen	*Lactobacillus, Bifidobacterium, Streptococcus, Lacto <10^5^*	RR(3)	1.28	(0.93, 1.77)
Chen	*Lactobacillus, Bifidobacterium, Streptococcus, Lacto >10^6^*	RR(3)	0.67	(0.34, 1.3)
Meng	*Lactobacillus, bifidobacterium*	SMD(2)	−0.12	(−0.61, 0.37)
Shi	*Lactobacillus*	SMD(6)	1.19	(0.46, 1.92)
Shi	*Lactobacillus Lacto <10^5^*	RR(2)	2.01	(1.42, 2.83)
Shi	*Lactobacillus Lacto >10^5^*	RR(2)	0.58	(0.27, 1.23)
Tovalino	–	SMD(6)	−0.78	(−1.65, 0.09)
Halitosis				
Huang	*OLP Lactobacillus, Weissella cibaria, Streptococcus,* *<* *4weeks*	SMD(5)	−0.58	(−0.87, −0.3)
Huang	*VSC Lactobacillus, Weissella cibaria, Streptococcus,* *<* *4weeks*	SMD(6)	−0.42	(−1, 0.17)
Huang	*OLP Lactobacillus, Weissella cibaria >4weeks*	SMD(2)	−0.45	(−0.85, −0.03)
Huang	*VSC Weissella cibaria >4weeks*	SDM(2)	−0.49	(−1.04, 0.06)
Valverde	*Total Weissella cibaria, Streptococcus*	SMD(4)	0.08	(−0.16, 0.31)
Yoo	*OLP Lactobacillus*	SMD(3)	−1.93	(−2.85, −1.02)
Yoo	*VSC Lactobacillus*	SMD(3)	−0.02	(−2.12, 2.07)
Yoo	*Total Lactobacillus*	SMD(6)	−0.98	(−2.25, 0.3)
Offenbächer	OLP	SMD(3)	1.85	(−0.77, 4.47)
Offenbächer	VSC	SMD(4)	2.29	(−0.64, −5.21)

LCI, lower confidence interval; UCI, upper confidence interval; RR, risk ratio; SMD, standardized mean difference; OLP, organoleptic; VSC, volatile sulphur compounds.

### The effects of probiotics supplementation on overall *Lactobacillus* count

Five eligible studies with 9 effect sizes [5 RR and 4 SMD], including 2,557 participants, examined the impact of probiotics supplementation on overall *Lactobacillus* count. Six of them demonstrated a significant improving effect on *Streptococcus* count. The age varied between 3 and 20 years. *Lactobacillus*, and *Bifidobacterium* were the predominant strains used for supplementation. The effect sizes of studies with CI are presented in Table 3.

### The effects of probiotics supplementation on halitosis

Four eligible studies with 10 effect sizes [10 SMD], including 1,957 participants, examined the impact of probiotics supplementation on halitosis. The age varied between 23 and 42 years. Studies investigated the effects of probiotics in volatile sulfur compounds (VSC) and organoleptic (OLP). *Lactobacillus*, *Weissella cibaria*, and *Streptococcus* were the predominant strains used for supplementation. The effect sizes of studies with CI are presented in Table 3.

### The effects of probiotics supplementation on dental caries (incidence/progression)

Three eligible studies with a total of 14 effect sizes (14 SMDs) examined the impact of probiotic supplementation on the progression of dental caries. Dental caries progression was assessed using standard clinical and radiographic measures, including changes in dmft/DMFT or dmfs/DMFS scores, the incidence of new carious lesions, or the progression of existing lesions during follow-up, as reported in the included meta-analyses. The studies included various probiotic strains such as *Lactobacillus*, *Bifidobacterium*, and *Streptococcus*, with specific analyses for *Lactobacillus* (*L.*) *paracasei* and *L. rhamnosus*. The standardized mean differences (SMD) demonstrated a significant reduction in caries progression when probiotics were administered. Pooled results showed SMDs of −0.24 (95% CI: −0.39 to −0.10) for mixed *Lactobacillus–Bifidobacterium–Streptococcus* combinations, −0.16 (95% CI: −0.33 to 0.01) for *L. paracasei*, and −0.41 (95% CI: −0.60 to −0.21) for *L. rhamnosus*. The detailed effect sizes and confidence intervals are presented in Table 3.

## Discussion

The present umbrella review aimed to evaluate the effectiveness of probiotics in managing oral health, focusing on halitosis, *Streptococcus* and *Lactobacillus* count, and tooth decay progression. The findings of this umbrella systematic review indicate that probiotic supplementation holds promise as an adjunctive strategy to improve oral health by modulating microbial markers, reducing halitosis-related outcomes, and slowing caries progression. Specifically, pooled analyses show that supplementation with probiotic strains specially *Lactobacillus*, *Bifidobacterium*, and *S. salivarius* species were associated with significant reductions in *S. mutans* counts, significant improvements in halitosis parameters like VSCs, organoleptic scores, and clinically meaningful reductions in caries progression. This pattern suggests a coherent beneficial effect of probiotics on oral ecological balance.

Several mechanisms have been proposed to explain how probiotics help oral health promotion. One possible explanation is that probiotics compete with *S. mutans* for adhesion sites on tooth surfaces, thereby hindering its colonization (5, 20, 21). In addition, many probiotic strains are known to secrete antimicrobial compounds that can suppress or inhibit the growth of *S. mutans* (22). Probiotics may also influence the host's immune system, enhancing the body's natural defenses against cariogenic bacteria (23). Numerous probiotic strains have shown direct antimicrobial and antagonistic effects against cariogenic and halitogenic microorganisms; for instance, *Lactiplantibacillus plantarum 14917* has been reported to suppress *Streptococcus mutans* growth, disrupt biofilm formation, and downregulate key virulence-associated genes in experimental models (4). Probiotics may reduce acidogenic and proteolytic activity in the oral microenvironment by altering biofilm composition and structure, thereby reducing demineralization, protease-mediated tissue breakdown and VSC generation (24). Furthermore, modulation of host response such as enhanced salivary IgA secretion, reduced pro-inflammatory cytokines, and improved mucosal immunity may disrupt creating an environment for dysbiosis and oral disease progression (25). Despite these promising mechanisms, further studies are required to clarify how probiotics exert their protective effects in oral cavity.

The present umbrella review's findings are broadly consistent with the conclusions of Beattie, 2024 study (26). In this comprehensive review, Beattie highlighted that specific *Lactobacillus* (particularly *L. rhamnosus* GG, *L. reuteri*, and *L. paracasei*) and *S. salivarius* K12/M18 strains exhibit the most robust evidence for reducing cariogenic and halitogenic microorganisms through direct antagonism, competitive adhesion, and modulation of host inflammatory responses. These mechanistic pathways mirror the trends observed in the present umbrella review, where the majority of meta-analyses demonstrated significant reductions in *S. mutans* and *Lactobacillus* counts and improvements in halitosis indices following probiotic supplementation.

Nevertheless, the present review has important limitations. There was considerable heterogeneity among the included meta-analyses in terms of probiotic strain identity, dosage, delivery vehicle (dairy, lozenge, tablet, rinse), population characteristics (age range 0.2 to 42.5 years), and intervention duration (7 days to 24 months). Such heterogeneity inevitably reduces the precision of pooled effect estimates and complicates interpretation and clinical translation because the efficacy of probiotic is strain-specific and dose-dependant. Further, many of the reported outcomes are surrogate markers (e.g., *S. mutans* counts, *Lactobacillus* counts, VSCs, organoleptic scores) rather than long-term clinical endpoints such as incidence of new cavitated lesions, progression of periodontal attachment loss or tooth loss. Additionally, while risk of bias assessment using AMSTAR-2 rated the majority of meta-analyses as moderate quality, the overall level of certainty in many outcomes remains low to moderate because of inconsistency, imprecision and indirectness. Finally, many studies had relatively short follow-up durations, limiting conclusions about the durability of probiotic effects after cessation of supplementation.

This umbrella systematic review offers a high-level summary of the documented effects of probiotics on important oral health outcomes, such as halitosis, bacterial counts, and caries-related metrics, by combining the data from several meta-analyses. The review makes cross-outcome comparison easier and aids in identifying trends and discrepancies in the available data by presenting results across outcomes within a unified framework. Information relevant to strains and doses is presented when available, but it does not suggest definite comparative efficacy; rather, it shows possible diversity in effects. Furthermore, the evaluation of bias risk is provided to put the results in context and improve openness about the caliber of the supporting data on oral ecology, while recognizing the shortcomings of the available data. Finally, the integration of mechanistic considerations with epidemiologic findings supports a more comprehensive interpretation of how probiotics may interact with the oral ecosystem, while acknowledging the limitations of the current evidence base.

In conclusion, while the evidence suggests that probiotic supplementation can favorably impact oral microbial markers, halitosis parameters and caries progression, the heterogeneity of interventions, reliance on surrogate endpoints and limited long-term data mean that recommendations for routine clinical use must remain cautious. Future research should emphasize well-designed randomized controlled trials with standardized probiotic strains and dosing regimens, long-term follow-up assessing hard clinical endpoints, and mechanistic sub-studies integrating microbiome, metabolome and host-response analyses. Such efforts will help to clarify optimal probiotic strategies for promoting oral health and validate their role as part of preventive dental care.

## Conclusion

Probiotic supplementation, especially selected strains of *Lactobacillus*, *Bifidobacterium* and *S. salivarius,* appears to have modest, potentially beneficial effects on cariogenic bacterial counts and halitosis measures and may slow caries progression in some populations. Nevertheless, the evidence base is constrained by heterogeneity in strains, doses and delivery forms, variable outcome definitions (surrogate vs. clinical endpoints), short follow-up in many primary trials, and only moderate methodological quality of the available meta-analyses. Until large, strain-specific randomized trials report consistent effects on clinically meaningful outcomes, routine use of probiotics for oral disease prevention should be considered experimental. Future research should prioritize greater methodological rigor and clinical relevance by (1) adopting standardized and validated outcome definitions and measurement methods, such as dmft/DMFT increments or ICDAS-based lesion staging for dental caries, calibrated organoleptic scoring and quantitative volatile sulfur compound assessment for halitosis, and harmonized microbiological sampling and analytical protocols; (2) preregistering study protocols to reduce selective reporting; (3) conducting adequately powered randomized controlled trials with sample sizes based on prespecified, clinically meaningful effect sizes and with sufficiently long follow-up durations (typically ≥12–24 months) to capture true caries incidence or progression; and (4) integrating mechanistic sub-studies, including oral microbiome and host-response analyses, to elucidate strain-specific and dose-dependent probiotic effects.
